# The International Medical Congress

**Published:** 1913-08-09

**Authors:** 


### THE INTERNATIONAL MEDICAL CONGRESS.

This week London has extended welcome to the members of the seventeenth International Congress of Medicine, who have come from the most diverse corners of the globe to take part in the proceedings. The contingents of medical men from the Continent and from the United States are exceptionally strong, both in numbers and in prestige. Some exceptionally interesting addresses have already been delivered and many valuable papers read; and there are plenty of good things yet to come before the closing meeting next Tuesday. In this connection an interesting analysis of the "reporters" to the various Sections and Sub-Sections has been published which sufficiently shows the catholicity of modern medicine and scientific research. The "reporters," it may be explained, are *savants* chosen to initiate discussions on certain subjects which are deemed of especial importance in the various Sections; they are authorities of eminence on their chosen topics, selected because they are recognised as such by their colleagues and because they command the confidence of the scientific world.

This analysis shows that of the two hundred and fifty-six "reporters," sixty are British, fifty-six German, forty-nine French, thirty-nine American, thirteen Austrian, eleven Italian, nine Swiss, four Dutch, three Hungarian, three Belgian, three Swedish, three Russian, two Danish, and one Japanese. Gratifying as it is to find our own country at the head of the list, it may yet be admitted that convenience of access is partly responsible for this preponderance. Still it does show that research is flourishing in Great Britain, notwithstanding the activity of those people who would like to see certain restrictions imposed which, in the opinion of scientists, would very seriously hamper progress and the advance of knowledge. And here we would draw attention to the fact that every single Section will, by the time these lines reach our readers, have considered on Thursday a resolution on Vivisection. We have every confidence that this resolution will have been passed unanimously by the Sections. It is condemnatory of the anti-vivisectionist agitation, and is to be regarded as a kind of international declaration of faith in this matter. In Lord Morley's address of welcome to the delegates at the Government banquet on Tuesday night he alluded briefly to this topic, though without touching on the simultaneous resolution which the different Sections have considered. Indeed, he skated over the ice so very gingerly that he might almost be suspected of views which would have been unacceptable to his audience. His allusions to the question of State interference in the problems of venereal disease were also very guarded; but at least they showed that the Government are taking notice of the request for a Royal Commission advocated in these columns last week.

Our voluntary hospitals and institutions are still, as they have long been, the admiration of the civilised world. We believe they can still bear comparison with any hospitals on the earth, and we are sure that those of them that are situated in London are being subjected to keen scrutiny by our distinguished visitors and guests. Several of them are making special arrangements for suitably receiving parties of foreign medical men and for showing them the methods and aims, not only of hospital organisation, but of British medicine and surgery as well. During the thirty-two years that have elapsed since the Congress last met in London hospital construction and management and, we may add, hospital life and manners have changed almost as rapidly as medicine and surgery. In personnel, too, there are but few left on the active staffs of our hospitals who held similar posts at the time of the last Congress. We are certain that our international guests will find much to take note of in the institutions they visit, and we hope they will ever retain pleasant recollections not only of the actual meetings of the Congress, but of their opportunities of watching our hospitals in being.

